# Completeness of *Neisseria meningitidis* reporting in New York City, 1989–2010

**DOI:** 10.1017/S0950268816000406

**Published:** 2016-03-17

**Authors:** L. ARAKAKI, S. NGAI, D. WEISS

**Affiliations:** New York City Department of Health and Mental Hygiene, Queens, NY, USA

**Keywords:** Capture–recapture analysis, meningitis, meningococcal disease, septicaemia, surveillance

## Abstract

Invasive meningococcal disease (IMD) completeness of reporting has never been assessed in New York City (NYC). We conducted a capture–recapture study to assess completeness of reporting, comparing IMD reports made to the NYC Department of Health and Mental Hygiene (DOHMH) and records identified in the New York State hospital discharge database [Statewide Planning and Research Cooperative System (SPARCS)] by ICD-9 codes from 1989 to 2010. Reporting completeness estimates were calculated for the entire study period, and stratified by year, age group, clinical syndrome, and reporting system. A chart review of hospital medical records from 2008 to 2010 was conducted to validate hospital coding and to adjust completeness estimates. Overall, 2194 unique patients were identified from DOHMH (*n* = 1300) and SPARCS (*n* = 1525); 631 (29%) were present in both. Completeness of IMD reporting was 41% [95% confidence interval (CI) 40–43]. Differences in completeness were found by age, clinical syndrome, and reporting system. The chart review found 33% of hospital records from 2008 to 2010 had no documentation of IMD. Removal of those records improved completeness of reporting to 51% (95% CI 49–53). Our data showed a low concordance between what is reported to DOHMH and what is coded by hospitals as IMD. Additional guidance to clinicians on IMD reporting criteria may improve completeness of IMD reporting.

## INTRODUCTION

Invasive meningococcal disease (IMD) is a serious condition caused by the bacterium *Neisseria meningitidis.* Although the incidence of IMD in the United States is relatively low at 0·25 cases/100000 persons [1], fatalities and sequelae remain high at 10–15% and 11–19%, respectively [2]. From 2000 to 2012 in New York City (NYC), the average annual incidence rate of meningococcal disease was 0·4 cases/100 000 person-years [3], but the case fatality (CF) was higher than national data. The IMD CFs for age groups 0–14, 15–24, 25–64, and ⩾65 years in NYC were 6%, 11%, 20%, and 31%, respectively (2000–2007, unpublished data), compared with national figures of 6%, 12%, 13%, and 24%, respectively, for the years 1998–2007 [4]. Prompt identification, treatment, and reporting of IMD to health departments are required to prevent further spread of disease [5].

To date, two studies have assessed the completeness of reporting of confirmed IMD to health departments in the United States. Ackman *et al.* [6] found completeness of IMD reporting to the New York State Department of Health (NYSDOH; excluding NYC) in 1991 to be 93%. In 2009, the Maine Department of Health and Human Services reported that completeness of IMD reporting from 2001 to 2006 was about 98% [7]. Both studies analysed health department surveillance and hospital discharge data, using the capture–recapture method to calculate completeness of reporting of confirmed cases [8, 9]. New York and Maine conducted extensive chart reviews and included only those cases in both systems that met confirmed and probable case definitions.

NYC's Health Code mandates immediate notification of clinically suspected as well as laboratory-confirmed cases of IMD. Prompt reporting and investigation of all cases is critical to prevent secondary cases and recognize clusters. The discrepancy between the NYC CF and nationally published figures has raised questions about differences in the epidemiology of IMD in NYC, among them the completeness of reporting. Underreporting of non-fatal, culture-negative cases could explain a higher than expected CF in NYC. To explore this hypothesis, we assessed completeness of IMD reporting to the NYC Department of Health and Mental Hygiene (DOHMH) by conducting a capture–recapture study. However, unlike the previous studies, we assessed the completeness of case reporting of all IMD reports, rather than limiting to only those with laboratory confirmation. From 1989 to 2005, only 4% of NYC IMD cases were culture negative. Since the DOHMH began routine use of polymerase chain reaction (PCR) testing for IMD in 2006, 19% of IMD cases have been culture negative (2006–2013) while the annual incidence of IMD has declined from 0·6/100 000 in 2000 to 0·3/100 000 in 2012 [3]. Our primary aim was to determine the completeness of reporting to DOHMH for all case definition categories of IMD (suspected, probable, confirmed). Our secondary aim was to determine if reporting completeness varied by patient's age, clinical syndrome, and reporting system (paper *vs*. electronic laboratory).

## METHODS

### NYC Communicable Disease Surveillance System

Upon receipt of a report of IMD, DOHMH staff conduct an investigation to confirm the case, identify close contacts, and facilitate the administration of antibiotic prophylaxis to prevent secondary transmission. All IMD reports originating from medical providers (including those determined as not meeting the case definition) in NYC residents from 1989 to 2010 appearing in the Communicable Disease Surveillance Database (CDSS) were included in this study. Fatal cases who were never hospitalized were excluded. Reports from non-sterile specimen sources (non-invasive cases) were also excluded. Case definitions for IMD used were based on the Council of State and Territorial Epidemiologists as employed by the National Notifiable Disease Surveillance System [10]. Changes to the case definition were made in 1997, 2005, and 2010. The case definition current at the time of IMD diagnosis was used to determine case status. To capture patients whose initial differential diagnosis included IMD but were subsequently diagnosed with a different reportable disease we included the following disease reporting categories in the match process: MEX (other bacterial meningitis), MAS (aseptic meningitis), PNE (*Streptococcus pneumoniae*), GBS (group B *Streptococcus*), GAS (group A *Streptococcus*), HIM (*Haemophilus influenzae*-causing meningitis), and HIX (*Haemophilus influenzae* not causing meningitis). Personal identifying information collected during investigations and entered into CDSS was used to match to the New York State (NYS) Statewide Planning and Research Cooperative System (SPARCS), which contains data on hospital discharges [11].

### SPARCS

Under NYS legislation (Section 28·16 of the Public Health Law), NYS hospitals are required to report inpatient and outpatient data to SPARCS. Data are anonymized and include patient demographics, treatments, procedures and diagnoses [12]. For this study, NYS hospital discharge data from 1989 to 2010 were obtained from SPARCS. Hospital records for NYC residents who were discharged from any hospital in NYS and coded as having IMD were identified using International Classification of Diseases, Ninth Revision (ICD-9) meningococcal disease codes 036·0–036·9 in any of the diagnosis fields. ICD-9 codes appearing in SPARCS are added by hospital billing departments upon review of the medical record and are used to justify patient evaluation, management, and procedure claims to third-party payers. To facilitate the matching process for this study, patient identifiers were requested from the NYSDOH, including date of birth, sex, address, hospital and medical record number, and discharge date. Full name and social security number were not available; however, starting in 1994, a unique personal identifier (UPI) variable was included, in conjunction with patient's sex, to link individual patient's records within SPARCS over time. The UPI comprises elements of the patient's first and last name and social security number.

### Matching protocol

Cases appearing in CDSS were matched to SPARCS data using an iterative process. A primary key using the same criteria as the UPI was created for patients in CDSS. The remaining records that could not be matched using the primary key were matched on combinations of other variables including date of birth, sex, medical record number and hospital name, patient's address, and discharge date. Extensive manual review was conducted of all matches to ensure accuracy. Review of randomly selected unmatched observations was also conducted to ensure that matches were not missed.

### Data analysis

Frequencies of matches were calculated for each year. Completeness of IMD reporting to DOHMH was calculated using the capture–recapture method for the entire study period and by year. SPARCS records that matched to non-meningococcal disease codes (MEX, MAS, PNE, GBS, GAS, HIM, HIX) were included; however, for SPARCS records that did not match we were unable to identify the CDSS cases in which a disease code may have changed from IMD to MEX, MAS, PNE, GBS, GAS, HIM, or HIX, and, therefore, should be included in the analysis as non-matches. Unreported IMD (Table 1, cell x) was estimated as the product of unique CDSS-only (Table 1, cell b) and SPARCS-only (Table 1, cell c) cases divided by the number of cases in both systems (Table 1, cell a); in other words, b × c/a. The estimated total number of IMD cases during the interval was the sum a + b + c + x. Completeness of reporting to DOHMH was calculated as the number of cases reported to CDSS divided by the estimated total number of cases in NYC (that could/should have been reported). Ninety-five per cent confidence intervals (95% CIs) for the total estimated number of IMD cases were calculated, and 95% CIs for completeness of reporting were calculated based on these estimates [6]. Additional completeness calculations were stratified by patient's age (<15 years, ⩾15 years) and by electronic clinical laboratory reporting system (ECLRS) implementation time period (pre-ECLRS, 1989–2001; voluntary ECLRS, 2002–2005; mandatory ECLRS, 2006–2010). Completeness estimates were also stratified by patient's clinical syndrome to determine if cases that presented with meningitis were more likely to be reported. CDSS cases were defined as having meningitis if there was any positive meningococcal test from cerebral spinal fluid or if the case was reported as meningitis. SPARCS meningitis cases were defined as those with ICD-9 codes for meningitis or encephalitis (036·0 and 036·1). All other cases were considered not to have presented with meningitis.

**Table 1.** Estimated number of reportable cases of invasive meningococcal disease in New York City, 1989–2010

		New York State Statewide Planning and Research Cooperative System (SPARCS)		
		Found	Not found	Total
New York City Communicable Disease Surveillance System (CDSS)	Reported	[a] 631	[b] 669	1300
	Not reported	[c] 894	[x] 948*	1842*
	Total	1525	1617*	3142* (95% CI 3007–3276)*

CI, Confidence interval.

* Estimate based on capture–recapture method.

[a] records found in both SPARCS and CDSS; [b] records found only in CDSS; [c] records found only in SPARCS; [x] estimate of unreported IMD.

It is important to note that capture–recapture methods are based on the assumptions that the data sources are independent, the data sources capture reports from the same population, individuals have the same probability of being captured by each source, matching is complete and accurate, and, in this case, all reports are true reports [6, 9]. Violation of these assumptions could result in the underestimation or overestimation of reporting completeness. Specifically, if a report has a higher probability of being captured by one source given its capture by the other source, the number of missing reports from either data source will be underestimated and the completeness of reporting will be overestimated. We performed a sensitivity analysis that estimates the highest proportion of completeness by calculating completeness of reporting assuming that no IMD cases were missed by both systems.

### *Post-hoc* chart review and analyses

We assumed that IMD-specific ICD-9 codes represented a clinical or laboratory indication of IMD. We conducted a hospital chart review of a subset of recent cases to determine the validity of our assumption. Chart reviews were conducted for SPARCS records with IMD ICD-9 codes and identifiable hospital medical record numbers using a standardized chart abstraction form for patients with discharge dates from 2008 to 2010. Charts were reviewed to determine whether there was either laboratory evidence or a physician's mention of IMD; cases with no clinical or laboratory indication of IMD were excluded from adjusted estimates. Completeness of reporting for 2008 to 2010 was recalculated adjusting for these cases and compared with an unadjusted completeness estimate for 2008 to 2010. The proportion of excluded cases based on chart review was applied to data for the entire study period, and an adjusted completeness for the whole study period was calculated to compare with the overall, unadjusted estimate. Completeness for 2008–2010 was also calculated restricting to cases that met confirmed or probable case definitions to allow comparison with previously published reports.

The study was approved by the NYSDOH Data Protection Review Board (application no. 1110-08).

## RESULTS

**Fig. 1.**
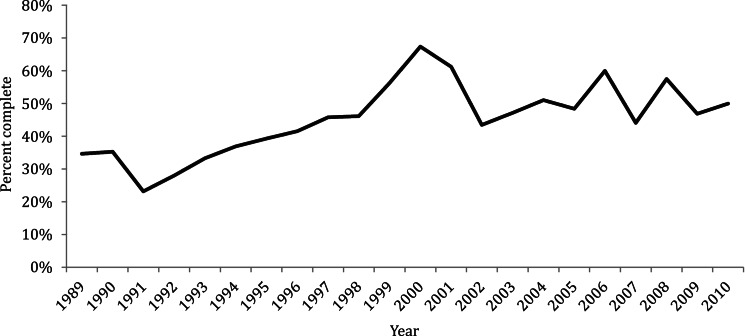
Estimated completeness of invasive meningococcal disease reporting to the New York City Department of Health and Mental Hygiene by year of diagnosis, 1989–2010.

**Fig. 2.**
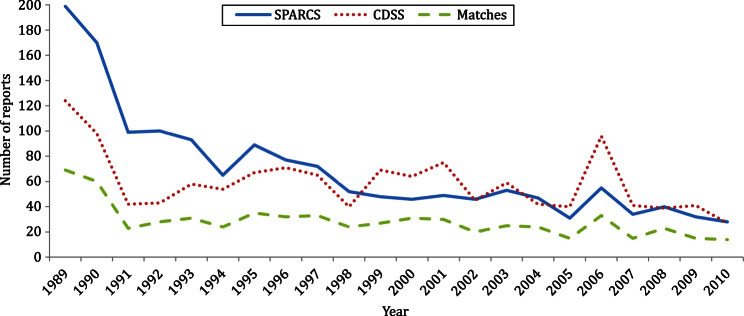
Invasive meningococcal disease reports found in the New York City Department of Health and Mental Hygiene communicable disease surveillance system (CDSS)*, the New York State hospital discharge database [Statewide Planning and Research Cooperative System (SPARCS)*], and in both data sources (matches) by year of diagnosis, 1989–2010. (* Numbers from individual data sources include records both matched and unmatched to the other data source.)

From 1989 to 2010, 1300 IMD cases in NYC residents were reported to CDSS. There were 1525 NYC residents identified in the SPARCS database with an IMD ICD-9 code. Of 2194 unique patients, 631 (29%) were present in both data sources. The estimated total number of case reports expected during the time period was 3142 (95% CI 3007–3276, Table 1). The unadjusted completeness of IMD reporting to DOHMH during the study period was estimated to be 41% (95% CI 40–43). Completeness estimates of IMD reporting over the entire interval are shown in Figure 1. The number of cases identified in CDSS, SPARCS, and both systems are shown in Figure 2.

**Fig. 3.**
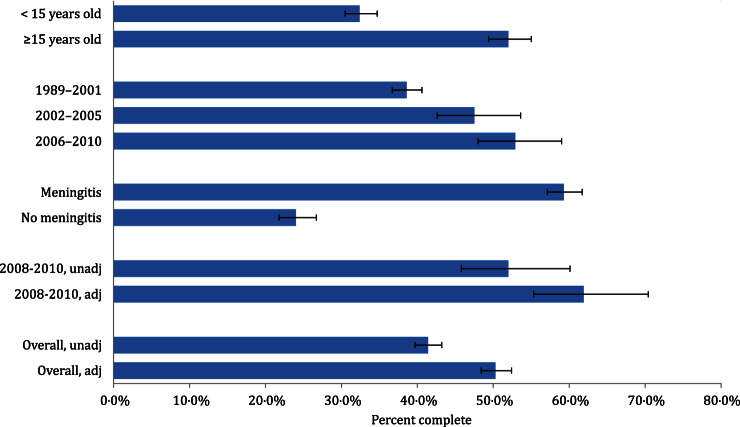
Overall and stratified completeness of reporting estimates for invasive meningococcal disease, in New York City, 1989–2010.

Completeness of reporting was 32% (95% CI 30–35) for patients aged <15 years and 52% (95% CI 49–55) for patients aged ⩾15 years (Fig. 3). In the period before ECLRS implementation (1989–2001), completeness was estimated to be 39% (95% CI 37–41). Completeness of reporting increased to 47% (95% CI 43–54) while ECLRS was voluntary (2002–2005), and to 53% (95% CI 48–59) when ECLRS became mandatory (2006–2010). The completeness of reporting for cases that presented with meningitis was 59% (95% CI 57–62), and 24% (95% CI 22–27) for cases without meningitis. The sensitivity analysis, under the assumption that the two systems captured all IMD cases, yielded a completeness of reporting for the entire study period of 59% (95% CI 56–63).

Between 2008 and 2010, there were 48 cases in the SPARCS database that could not be matched to CDSS and were selected for chart review. Of these, 45 hospital charts were available for review. None of the charts contained laboratory evidence of *N. meningitidis*. Additionally, in 16 (33%) records there was no physician documentation that IMD was considered as a diagnosis. These cases were determined to have been incorrectly assigned an IMD ICD-9 code and were excluded from the adjusted estimates. The three charts not available for review were included in the adjusted estimates. The adjusted completeness of reporting for 2008–2010 improved from 52% (95% CI 46–60) to 62% (95% CI 55–70). Extrapolating the estimate that 33% of cases in SPARCS were miscoded to data for the entire study period improved the completeness of reporting to 51% (95% CI 49–53, Table 2). There were 71 confirmed IMD cases, six probable cases, and 30 individuals determined not to be cases from 2008 to 2010 in CDSS. The SPARCS registry does not contain laboratory information so the corresponding proportions of confirmed and probable cases were not obtainable. When we limited cases to the 77 confirmed and probable cases from CDSS, the completeness of reporting increased to 94% (95% CI 90–98).

**Table 2.** Estimated number of reportable cases of invasive meningococcal disease in New York City adjusted for ICD-9 coding errors, 1989–2010

		New York State Statewide Planning and Research Cooperative System (SPARCS)		
		Found	Not found	Total
New York City Communicable Disease Surveillance System (CDSS)	Reported	[a] 631	[b] 669	1300
	Not reported	[c] 599	[x] 635*	1234*
	Total	1230	1304*	2534* (95% CI 2434–2632)*

CI, Confidence interval.

* Estimate based on capture–recapture method.

[a] records found in both SPARCS and CDSS; [b] records found only in CDSS; [c] records found only in SPARCS; [x] estimate of unreported IMD.

## DISCUSSION

We performed an evaluation of the completeness of *N. meningitidis* reporting using the capture–recapture method. Unadjusted and adjusted (for ICD-9 coding mistakes discovered in the *post-hoc* analysis) estimates of completeness were lower than European studies that only evaluated confirmed cases over a similar time period [13, 14]. De Greef *et al*. [15] found the completeness of IMD reporting to the notifiable disease surveillance system in The Netherlands for the years 1993–1998 to be 49%, comparable to our findings before the implementation of ECLRS. The low finding was attributed to false-positive diagnoses in both the mandatory reporting and hospital discharge systems. Our limited chart review of cases not reported to CDSS failed to find any probable or confirmed IMD cases, and in 33% of cases, the physicians did not suspect IMD, suggesting erroneous ICD-9 coding. The remaining 29 charts contained some indication of IMD and, thus, those cases should have been reported to DOHMH as suspect IMD. Additionally, we identified 31 confirmed IMD cases in CDSS that could not be matched to SPARCS, suggesting coding omissions despite clear laboratory evidence of IMD. Since testing for *N. meningitidis* by PCR is only performed when DOHMH is notified of IMD, we cannot assuredly state that no additional cases occurred during the period.

The trend in increasing completeness of IMD reporting paralleled the transition to ECLRS; however, it is unlikely that the increase from 39% reporting pre-ECLRS to 53% after full implementation is a direct result of this transition. While ECLRS may have marginally increased the reporting of culture-positive cases, suspected, probable, and confirmed IMD cases are required by the NYC Health Code to be immediately reported by phone. The apparent improvement in completeness could be explained by the decline in IMD incidence. With fewer actual IMD cases, hospitals may have reduced incorrect ICD-9 coding.

Completeness of reporting also varied by patient's age and IMD syndrome. As expected, reporting of IMD cases with symptoms of meningitis, a more recognizable presentation of IMD, was better than those presenting with the protean symptoms of bacteraemia. The entity of fever and rash is well described in children and may represent occult bacteraemia, prompting clinical staff (including nurses) to consider IMD in their differential diagnosis, which in turn triggers ICD-9 coders to include it in the list of admission or discharge diagnoses [16]. Our chart review did not examine nursing notes for mention of IMD. The rapid administration of antibiotics may render culture-based tests negative, and while the absence of laboratory confirmation may explain the failure to notify public health officials, our experience using PCR for culture-negative cases suggests that this does not rule out *N. meningitidis* as the aetiological agent.

While the sub-analysis for confirmed and probable IMD cases found high completeness of reporting to DOHMH at 94%, comparable to previous US studies on completeness of IMD reporting [6, 7], IMD surveillance requires public health to cast a wider net beyond laboratory-confirmed cases. We believe that including all reported cases in our analysis produced a more accurate assessment of the completeness of IMD reporting than only including confirmed cases. In the past decade, NYC has had two large IMD outbreaks that prompted resource-intensive vaccination campaigns [17, 18]. Whether unreported IMD cases played any role in the propagation of the outbreaks cannot be determined; however, during the outbreak years of 2006 and 2012–2013, 26% of IMD cases were diagnosed by PCR and other non-culture-based methods. Timely reporting of IMD cases to public health officials remains critical to control efforts. The identification and rapid prophylaxis of close and household contacts relies on immediate and thorough reporting of suspected as well as laboratory-confirmed IMD cases. The aggressive use of PCR has allowed DOHMH to make the diagnosis of IMD in a substantial proportion of culture-negative cases that previously could have been missed. Additionally, complete reporting is important to recognize links among cases that might lead to identification of clusters or outbreaks, evaluate the effectiveness of vaccination, and accurately tally the burden of disease. The latter is necessary for provider education, assessment of subpopulations at greatest risk, and resource allocation.

Our evaluation of completeness of IMD reporting is subject to several limitations including possible violations of the major assumptions of the capture–recapture method: (*a*) individuals in the two systems can be confidently matched; (*b*) individuals in each system have the same chance of being captured; and (*c*) the two systems are independent [19]. Individuals diagnosed outside of NYC, but in NYS, are reported to DOHMH and would have the same chance of appearing in both systems. We removed individuals from CDSS who were diagnosed in other states and, therefore, would not have had the opportunity to be captured in SPARCS. Sufficient identifying information existed within each file ensuring confidence in our ability to identify and correct any matching errors. The assumptions of equal chance of capture and independence of data sources are often the greatest concerns when using the capture–recapture method to analyse epidemiological data [9]. In our study, however, the sources of data arise from separate and distinct healthcare functions [13]. Surveillance reports arise from the clinical evaluation of patients from hospital personnel who are required to report suspected, probable, and confirmed cases of IMD. Entry into the SPARCS database originates from ICD-9 codes added during the billing review process. While we believe that individuals with confirmed, probable, and suspect IMD have similar probabilities of being captured by both systems, CDSS and SPARCS are not entirely independent. ICD-9 coders utilize information contributed to the medical record by clinicians and laboratories. This positive dependency would likely result in an underestimate of the total magnitude of IMD cases, which would have inflated our completeness of reporting estimate [8]. Case severity and age may influence the likelihood of an individual being reported to DOHMH; however, we stratified by age and IMD syndrome to account for this potential bias. Given that the implication of the assumption violation is that we may have underestimated the number of unreported cases, the conclusions developed are still relevant. The completeness of case reporting may actually be lower than estimated.

It is also conceivable that ICD-9 miscoding was not consistent over the entire interval. We were not able to perform extensive chart reviews and, therefore, cannot be confident of our extrapolation of the proportion of miscoded IMD hospital records for the entire study period. We were also not able to identify CDSS cases that were initially reported as IMD, but had a change in disease code and did not match in SPARCS. Inclusion of false matches would have also served to overestimate our completeness of reporting.

Finally, we also could not control for numerous other factors during the long study period which may have influenced IMD reporting over time, such as changes in ICD-9 coding and clinical practice. In addition to improvements in diagnostic testing, ECLRS, and the declining incidence of IMD, routine childhood vaccine recommendations were introduced in 2005 [2], and there has been an expansion in the use of electronic medical records. Our evaluation did not attempt to dissociate the effects that these events may have had on the results.

## CONCLUSION

Our findings suggest that DOHMH does not receive all reports of IMD, specifically on children aged <15 years. Chart review findings revealed no additional probable or confirmed cases and ICD-9 coding discrepancies between SPARCS data and hospital chart records, as well as CDSS data. We believe there is a need for health departments to provide additional guidance to clinicians on reporting criteria for IMD, in particular for presentations other than meningitis and those instances where culture confirmation is unlikely.

## References

[1] Centers for Disease Control and Prevention. Summary of notifiable diseases – United States, 2011. Morbidity and Mortality Weekly Report. Surveillance Summary 2013; 60: 1–117. 23820934

[2] Centers for Disease Control and Prevention. Prevention and control of meningococcal disease: recommendations of the Advisory Committee on Immunization Practices (ACIP). Morbidity and Mortality Weekly Report. Recommendations 2013; 62: 1–30. 9048846

[3] New York City Department of Health and Mental Hygiene. Epiquery: NYC Interactive Health Data System – Communicable Disease Module, 2000–2012 (http://nyc.gov/health/epiquery). Accessed February 2015.

[4] Cohn AC, Changes in *Neisseria meningitidis* disease epidemiology in the United States, 1998–2007: implications for prevention of meningococcal disease. Clinical Infectious Diseases 2010; 50: 184–191. 20001736 10.1086/649209

[5] MacNeil J, Cohn A. Chapter 8: meningococcal disease. In: Roush SW, Baldy LM, eds. *Manual for the Surveillance of Vaccine-Preventable Diseases*, 2011 (http://www.cdc.gov/vaccines/pubs/surv-manual/chpt08-mening.html#f1).

[6] Ackman DM, Birkhead G, Flynn M. Assessment of surveillance for meningococcal disease in New York State, 1991. American Journal of Epidemiology 1996; 144: 78–82. 8659488 10.1093/oxfordjournals.aje.a008857

[7] Centers for Disease Control and Prevention. Completeness and timeliness of reporting of meningococcal disease – Maine, 2001–2006. Morbidity and Mortality Weekly Report 2009; 58: 169–172. 19247263

[8] Hook EB, Regal RR. Capture-recapture methods in epidemiology: methods and limitations. Epidemiologic Reviews 1995; 17: 243–264. 8654510 10.1093/oxfordjournals.epirev.a036192

[9] Tilling K. Capture-recapture methods – useful or misleading? International Journal of Epidemiology 2001; 30: 12–14. 11171841 10.1093/ije/30.1.12

[10] Centers for Disease Control and Prevention. National notifiable disease case definition, meningococcal disease 2010 (http://wwwn.cdc.gov/NNDSS/script/casedef.aspx?CondYrID=774&DatePub=1/1/2010). Accessed June 2011.

[11] New York State Department of Health. Statewide Planning and Research Cooperative System (SPARCS), 1989–2010 (data update: April 2012).

[12] New York State Department of Health. Statewide Planning and Research Cooperative System (SPARCS), SPARCS Operational Guide (https://www.health.ny.gov/statistics/sparcs/training/docs/sparcs_operations_guide.pdf). Accessed 3 December 2015.

[13] Berghold C, Invasive meningococcal disease in Austria 2002: assessment of completeness of notification by comaprison of two independent data sources. Wiener Klinische Wochenschrift 2006; 118: 31–35. 10.1007/s00508-005-0502-0 16489523

[14] Horwitz MF, Samuelsson S, Molbak K. Declining incidence of meningococcal disease in Denmark, confirmed by a capture-recapture analysis for 1994 and 2002. Epidemiology and Infection 2008; 136; 1088–1095. 17892628 10.1017/S0950268807009466 PMC2870894

[15] de Greef SC, Underreporting of meningococcal disease incidence in the Netherlands: results from a capture-recapture analysis based on three registration sources with correction for false positive diagnoses. European Journal of Epidemiology 2006; 21: 315–321. 16685583 10.1007/s10654-006-0020-z

[16] Baker RC, Fever and petechiae in children. Pediatrics 1989; 84: 1051–1055. 2587134

[17] Weiss D, Epidemiologic investigation and targeted vaccination initiative in response to an outbreak of meningococcal disease among illicit drug users in Brooklyn, New York. Clinical Infectious Diseases 2009; 48: 894–901. 19231975 10.1086/597257

[18] Kratz MM, Community-based outbreak of *Neisseria meningitidis* serogroup C infection in men who have sex with men, New York City, New York, USA, 2010–2013. Emerging Infectious Diseases 2015; 21: 1379–1386. 26197087 10.3201/eid2108.141837 PMC4517726

[19] International Working Group for Disease Monitoring and Forecasting. Capture-recapture and multiple-record systems estimation I: history and theoretical development. American Journal of Epidemiology 1995; 142: 1047–1058. 7485050

